# Frigate: A Novel Dataset Comprising Wide Field-of-View Astronomical FITS Images of Low Earth Orbit Scenes for Machine Learning Applications

**DOI:** 10.1038/s41597-025-06220-0

**Published:** 2025-11-28

**Authors:** Daniel S. Roll, Zeyneb Kurt, Yulei Li, Ralph Dinsley, Wai Lok Woo

**Affiliations:** 1 Northumbria University, College Street, Newcastle-upon-Tyne, NE1 8ST England; 2 University of Sheffield, The Wave, 2 Whitham Road, Sheffield, S10 2TN England; 3 3S Northumbria Ltd., Bondgate Without, Alnwick, NE66 1PR England; 4 ExoAnalytic Solutions Inc., Foothill Ranch, California 92610 USA

**Keywords:** Scientific data, Databases, Solar physics

## Abstract

This report presents Frigate, a novel astronomical dataset comprising still-frame images of a section of Low Earth Orbit (LEO), collected by ExoAnalytic Solutions Inc., a commercial space situational awareness (SSA) provider using ground-based optical systems. Datasets of this kind are rare and typically inaccessible to most stakeholders. Through agreement with the company, the images were collected and prepared for use in machine learning and computer vision tasks related to SSA. The raw images contain lens glare artefacts, vignetting, and outer-region degradation. To address this, we additionally release a lightly processed version of each image featuring artefact removal, background subtraction, and sharpening. Usage notes describe optional downstream techniques including stacking, streak detection, and automated annotation. Given the lack of publicly available space surveillance data, this dataset represents a valuable resource for satellite tracking, orbital debris detection, and astronomical computer vision. The dataset described in this paper is wholly owned by ExoAnalytic Solutions Inc. and was released to the research team through mutual agreement of the benefits of its processing and application to the domain. All data and code are openly available.

## Background & Summary

In the modern era, the large volume of available data across all domains has become a cornerstone of progress for investigation in such fields, resulting in significant advancements in science, technology and policy for both academic and industry purposes^1^. In the past, scientific research often progressed slowly, as researchers relied on scarce datasets that regularly had to be gathered or compiled first-hand and were in many cases incomplete or incompatible; a stark contrast to the petabytes of publicly available data that can be found today using simple internet search engines, fundamentally changing the way research is conducted as a whole^2^. Around the turn of the century, the term ‘big data’ was coined to describe the ever-increasing amount of data being rapidly generated from various sources, with Chen and Zhang^3^ explaining this as large amounts of complex data that cannot be managed efficiently, even by state-of-the-art technologies. The advent of big data has undoubtedly revolutionised many fields of research, as the sheer volume of information allows for myriad angles of investigation and increases the chances of identifying patterns or correlations that would be more challenging to recognise in more limited circumstances. Thus, whilst the sheer volume of such data may be challenging to evaluate, correct application and usage of it offers new insights into many problems and strives to push innovation forwards. With the crucial role data plays in research, ensuring it is of high quality is necessary as only correct data can truly provide valid, resilient insights into the underlying field^4^. High-quality data ensures both accuracy and replicability as well as reliability in the analysis, which in turn allows researchers to draw informed and meaningful conclusions from their investigation. Furthermore, good data minimises errors and therefore any bias, improving overall confidence in the results. With contemporary data analysis employing complex methods such as machine learning - which is often applied in equally complex fields such as healthcare or automation - the need for high quality data is amplified, as the resultant inferences may have significant importance for end users, and it is relatively easy for models to ‘learn’ incorrect concepts during training as a result of the data^5^. Overall, the impact of data selection and quality on scientific inquiry is profound, and carefully curating datasets is a must for advancing knowledge across all research domains.

With the introduction of the open data movement, which was introduced by initiatives such as the Open Knowledge Foundation and Open Data Institute in the early 2000s^6^ and advocates for freely accessible and shareable data, research has been heavily democratised. Both governments and companies began to open up their previously protected datasets for public use amid rising concerns of the importance of such data on problem solving. There is now a much broader scope for collaboration in the modern era, and the transparency of research is across scientific disciplines has greatly improved over time. Many datasets are submitted for open access across a range of repositories such as GitHub or Kaggle, with most developed search engines already optimised to find relevant results^7^, allowing researchers to pick and choose data that suits their individual goals. However, the amount of available data varies heavily, conditional to the research domain. In fields such as health or social sciences, there are many easily-accessible public datasets that can be downloaded relatively straightforwardly from a range of online repositories, which are often validated and maintained by large groups of researchers, ensuring their authenticity is substantiated. For more niche topics, it becomes much more difficult to curate data either due to lack of availability or lack of access. This is especially true in fields with significant commercial overlap, which may affect availability of data^8^, or significant government or defence overlap, which may impact access to such data^9^.

One such field in which appropriate datasets are sparse is the research domain of Space Situational Awareness (SSA)/Space Domain Awareness (SDA). This area of research is broadly focused on detecting and tracking orbital objects, such as satellites or debris, as to improve the knowledge of the stellar landscape in order to be able to safely launch spacecraft or make on-orbit manoeuvres^10^. As outlined in the relevant literature, improving modern SSA practices is crucial due to the increasing risk of the Kessler Syndrome - a scenario where cascading collisions of orbital objects could occur, underscoring the importance of the thorough analysis and cataloguing of such objects to prevent an exponential increase in risk^11^. Over the decades, human efforts to explore and innovate in space have resulted in the accumulation of debris in Earth’s orbit. This includes a variety of debris material, sizes and origins, the majority originating from discarded components of previous space missions, with only a small fraction comprising active satellites or spacecraft. The presence of these objects poses significant hazards to the future of space exploration and travel, due to the attributed lethal risk of collisions and thus, potentially generating additional debris from impacts. Failure to manage this debris could not only delay future space missions but also endanger the sustainability of space activities^12^. Resolving the existing debris landscape, therefore, as well as limiting future residuals, is essential to ensure the future of space exploration. One contemporary SSA method, moving towards debris removal, is using AI to estimate the positions of objects. Literature describes many evaluated models for object detection and tracking over the years such as CNN, KPCA and MAML^13–15^ with various data such as light-curve estimations, radar scans and synthetic RGB images^16–18^. With the growth and successful application of object detection to many fields such as robotics^19^ and manufacturing^20^, there are examples of research now applying similar technologies such as YOLO to the SSA domain^12,21,22^. Luca *et al*.^23^ explains that for such approaches, the quality of the training data is paramount; high-quality image datasets stand to provide the model with sufficient input as to correctly extract features, improving detection capabilities and supporting the validity of their outputs. For SSA object detection and tracking, ideal data often comprises wide-field-of-view (WFOV) images captured with ground-based optics over a certain period of time. Whereas, as previously mentioned, relevant data may be in abundance in other fields, for such SSA activities there seems to be a significant lack of publicly accessible datasets that are readily available. That is not to say there is no data at all - there are datasets such as the ESA’s Kelvins competition dataset^24^ as well as many smaller subsets of images available through online platforms such as Github or Kaggle, although they are comparably few and far between in a field with such high amounts of data production and collection. This can be explained, however, due to the significant commercial and governmental/defence overlaps with SSA research. First and foremost, there is a dual-use nature for SSA-based detection; technology that can be used to locate objects for research purposes could also be used for defence purposes, nefarious or otherwise. Thus, whilst object catalogues are available, they are often not true representations of the scale or number of objects that physically occupy an area of space as many covert or blacklisted satellites will undoubtedly be redacted for obvious reasons. Access to high quality images referencing the full unabridged catalogues will often be limited to first-hand defence personnel, meaning a lot of data is not publicly accessible and therefore cannot be used for research purposes. Furthermore, many commercial SSA companies offer object tracking as a product or service and many have access to market leading high-quality image data gathered to inform their relevant technologies. Understandably, from a commercial standing it does not make sense to make this data publicly available, and it therefore also remains inaccessible for research purposes. As much of the desirable image data is collected and owned by either government or commercial entities, researchers seldom gain access to such images and therefore are forced to rely on the previously mentioned sparsely available public datasets for the development of new SSA technologies and approaches.

Clearly, the development of modern data-driven methods has catalysed progress across numerous scientific domains, with machine learning and artificial intelligence (AI) technologies enabling automation and inference in increasingly complex environments. However, such advancements are inherently dependent on the quality and availability of data, especially when applied to safety-critical domains such as Space Situational Awareness (SSA). In our prior work^25^, we introduced a framework that combines multimodal large language models (LLMs) with graph-based retrieval-augmented generation (GraphRAG), specifically targeting the application of orbital debris detection and explanation. The research highlighted the potential for language-and-vision models such as LLaVA, augmented by structured graph databases (Neo4j) to deliver interpretable, image-grounded responses to SSA-related imagery. A key limitation acknowledged in that work, however, was the reliance on synthetic images. Public access to real-world astronomical image sequences remains limited, particularly in the context of satellite detection, orbital debris monitoring, and space domain awareness (SDA). Most available datasets rely on synthetic data or curated examples from narrow observational windows, making it difficult for researchers to evaluate temporal or object-tracking algorithms on representative data. This release describes a previously unseen, novel astronomical dataset, gathered by a leading commercial SSA entity, ExoAnalytic Solutions Inc. for use in downstream research tasks. The dataset, codenamed Frigate, was provided through collaboration with ExoAnalytic Solutions Inc., and comprises wide-field-of-view (WFOV) images captured using commercial ground-based optical sensors. Initial processing steps were undertaken to enable clearer reuse. These include median-based background subtraction, artefact removal, and Gaussian sharpening. The resulting enhanced images are included alongside the raw files to provide flexible entry points for future workflows. To our knowledge, Frigate represents one of the few SSA datasets made publicly available for research purposes. The publication of this dataset therefore constitutes a significant contribution to the field, addressing the well-documented scarcity of open-access orbital imaging data and enabling new experimentation in object detection, tracking, and many other research areas.

## Methods

### Data Acquisition

ExoAnalytic Solutions Inc. are an American SSA company based in Foothill Ranch, California specialising in both aerospace and mission-system technology solutions. The company focuses on providing space domain awareness through a large network of sensors and advanced tracking systems, boasting the largest global network of optical telescopes, the ExoAnalytic Global Telescope Network (EGTN). The EGTN provides real time data to support commercial, as well as defence and intelligence operations, with a focus on tracking and monitoring satellites, debris and other orbital objects. Recently, the company has expanded to begin establishing a presence in the UK, through its acquisition of 3S Northumbria Ltd., a British SME specialising in sustainable space solutions with a focus on space situational awareness. The company promotes the idea of a circular economy in space, advocating for the sustainable usage of space resources and technologies designed to both reduce orbital debris and ensure long-term space sustainability. This is expected to enhance the availability of commercial SSA capabilities in the UK, leveraging 3S Northumbria’s innovative technology and expertise to support government and commercial space initiatives.

**Fig. 1:**
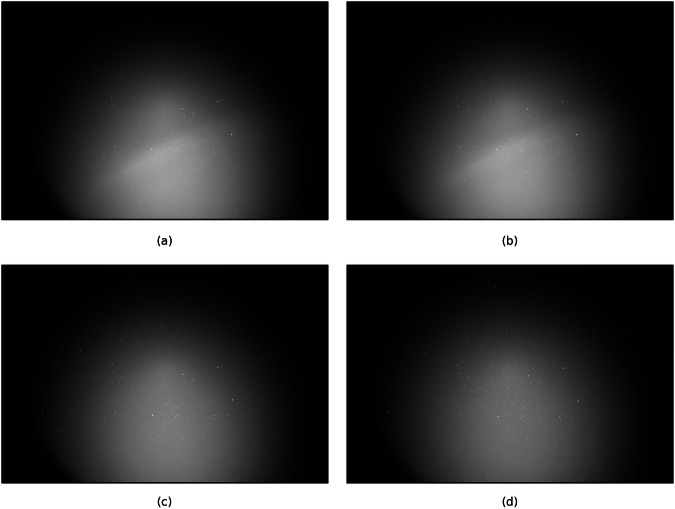
Sample raw frames from the *Frigate* dataset: (**a**) Capture_00069 02_55_59Z.fits, (**b**) Capture_00088 02_56_08Z.fits, (**c**) Capture_00243 02_58_41Z.fits, (**d**) Capture_00317 03_00_14Z.fits.

The raw data^26^ were gathered by ExoAnalytic Solutions Inc. using a commercial-grade ground-based optical telescope system. Each sequence of FITS-format still frames captures a continuous segment of the night sky in which low Earth orbit (LEO) activity may be present. The imaging system comprises a QHY600M PCIe CMOS monochrome camera with a sensor resolution of 9600 × 6422 pixels. The sensor has square pixels with a physical size of 3.76*μ*m, and the optical configuration uses a 28mm focal length lens at an f/1.4 focal ratio, yielding a wide field of view (WFOV) suitable for space surveillance applications. Exposure time was fixed at 0.5 seconds per frame, providing a high temporal resolution across sequences. Each image is stored as a 16-bit integer array using the FITS format, with unsigned pixel values represented via standard FITS rescaling parameters (BSCALE = 1, BZERO = 32768). All metadata are retained in the FITS headers, including timestamps (DATE-OBS), binning settings (XBINNING, YBINNING = 1), and camera software configuration (SharpCap v3.2.6377.0). The raw data comprises 2000 FITS files, examples of which can be seen in Fig. 1.

### File Metadata

Each raw frame in the Frigate dataset^26^ is provided as a 16-bit grayscale image in the Flexible Image Transport System (FITS) format. Metadata embedded in the header of each FITS file provides observational parameters, instrumental configuration, and acquisition timing. An example header, from file Capture_00001 02_55_24Z.fits, is shown below:

SIMPLE = T / C# FITS: 04/01/2024 19:55:25

BITPIX = 16

NAXIS = 2 / Dimensionality

NAXIS1 = 9600

NAXIS2 = 6422

EXTEND = T / Extensions are permitted

GAIN = 0 /

DATE-OBS= '2024-04-02T02:55:24.3834903' / System Clock:Est. Frame Start

SWCREATE= 'SharpCap' / v3.2.6377.0, 64 bit

CCD-TEMP= 0 /

XBINNING= 1 /

YBINNING= 1 /

XPIXSZ = 3.76 / [*μ*m] Pixel size in X

YPIXSZ = 3.76 / [*μ*m] Pixel size in Y

EXPTIME = 0.5 / [s] Exposure time

INSTRUME= 'QHY600MPCIE' / Camera model

FOCALLEN= '28.0 / [mm] Focal length'

FOCRATIO= '1.4 / Focal ratio'

BZERO = 32768

BSCALE = 1

Key header fields are as follows:

- BITPIX, NAXIS, NAXIS1/2: Indicates 2D image structure with 16-bit signed integers across a 9600 × 6422 pixel resolution.
- EXPTIME: 0.5s integration per frame, providing sub-second temporal resolution across each scene.
- DATE-OBS: Timestamp for the start of exposure, consistent with local system clock (UTC).
- XPIXSZ, YPIXSZ: Square pixel geometry of 3.76 *μ*m; when combined with the focal length (28mm), this implies an approximate pixel scale of 27.7 arcsec/pixel, suitable for WFOV surveillance.
- INSTRUME, CCD-TEMP, GAIN: Identifies the imaging sensor (QHY600M PCIe), though GAIN and CCD-TEMP were unset (0) in this configuration and should be treated as uncalibrated.
- BZERO, BSCALE: Standard FITS rescaling fields, indicating unsigned pixel range with values offset by 32,768 (i.e., true values = data + 32768).

These fields provide sufficient information to interpret the raw values for display or scientific analysis. Users should note that no flat-field or dark frames were provided, and header fields such as gain and temperature are default values. As such, photometric calibration is not feasible without assumptions, but spatial and temporal characteristics are fully retained.

## Initial Data Processing

In order to aid users in utilising the data for advanced machine learning techniques such as computer vision, a simple preprocessing pipeline was applied to the raw frames, producing a cleaned PNG files that are ready to use in multiple applications. Ravi and Basavaprad^27^ explain the importance of correct image selection and preparation for such applications, as high-quality image data is essential for optimising machine learning technologies. Good data should have a high resolution, minimal noise and constant lighting conditions to ensure accurate model training and prediction, with good image quality enabling the extraction of pertinent features that are essential for tasks such as object detection, classification and image segmentation. Conversely, poor image quality can lead to risks such as distorted models, imprecise predictions and reduced generalisation capabilities. Problems such as blurred images, inconsistent labelling or insufficient diversity of the dataset can lead to overfitting, where a model performs well on training data but cannot be generalised to new data.

All data processing was completed using Python 3. Initially, the data was accumulated and saved locally before being loaded for processing. In order to visualise the images, the FITS files were loaded and opened, utilising the popular *astropy* library, a comprehensive Python library that offers tools for performing common file operations for astronomical data^28^. This was used to load both the files and associated metadata, retrieving information that was used to compute the mean and standard deviation of the pixel values, as to set normalisation boundaries and improve the contrast of the images. Using the boundary values, a linear normalisation was applied as to adjust the range of pixel intensity values and, in turn, improve the visibility of any features^29^. The images were then displayed using the *matplotlib* library, resulting in images such as the examples shown in Fig. 1.

**Fig. 2:**
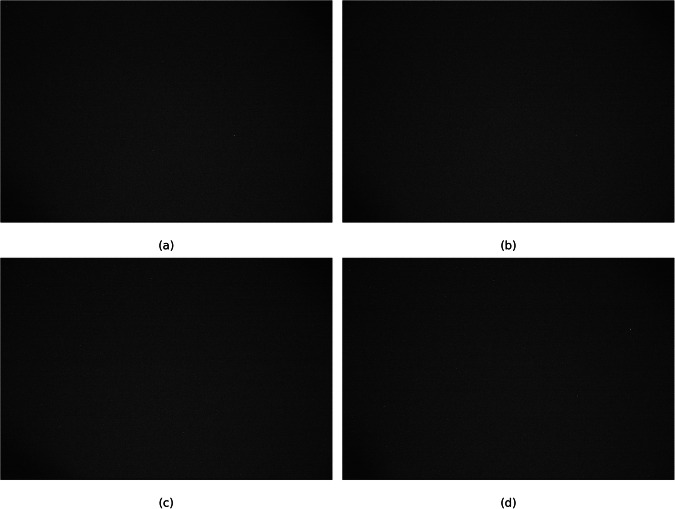
The sample frames after the background subtraction process: (**a**) Capture_00069 02_55_59Z.fits, (**b**) Capture_00088 02_56_08Z.fits, (**c**) Capture_00243 02_58_41Z.fits, (**d**) Capture_00317 03_00_14Z.fits. The frames are distorted or 'fuzzy' at this stage of the processing as sharpening steps have not been applied yet.

Upon inspection of the processed images, although displaying correctly, there are a number of important observations. Firstly, there is a large lens artefact - in this incidence, a lens flare - spanning the majority of the images. Artefacts such as this occur when the optical components of the telescope or camera introduce slight distortions, due to reflections from light sources such as the sun, or simply lens misalignment^30^. Furthermore, the intensity of the light reflection in the centre of the artefact makes distinguishing any proximate visual information difficult, which has obvious ramifications for any computer vision technologies that rely on detecting pixel intensity disparities. Finally, there appears to be a significant lack of visual information in the darker corners of the images, which logically should also show dense star clusters. This, however, may be explained by vignetting caused by the presence of the aforementioned artefact. Reflecting on these observations, it is evident that removing the lens artifact would be paramount for successfully preparing the dataset. Typical astronomical data processing has a number of important steps that are used to rectify such visual issues, notably background subtraction and flat-field multiplication. Background subtraction is a process where the variance in observable background light, such as that experienced by skyglow or light pollution, is removed from the image^31^. This is usually achieved by creating a light model, in which the background noise is averaged over the frames to give a representation of the variance which is then subtracted from each image; thus, the fluctuations in visibility and intensity of the light are removed. For the current dataset, no background light model was provided and therefore had to be completed early in the processing stage. The averaging process was applied on a rolling window basis - for each frame, the median average of the background for the ten adjacent frames was calculated and then subtracted from the image. This allowed for the removal of the irregularities in lighting whilst allowing the larger temporal differences, such as those relating to the sun’s position, to only affect the frames in which such lighting would be appropriately experienced. Thus, the background model for a frame would give a truer representation of the background conditions at that particular time step. When applied to the raw images, the process appeared to correctly remove fluctuations in background intensity, however the images appeared overly grey and washed out. A clipping step was then added to ensure all pixel values were non-negative, and the visualisation percentiles were adjusted to modify the upper and lower boundaries for the display range, excluding any extreme pixel values that may have been skewing the visualisation of the data. The product of this processing step can be seen in Fig. 2.

**Fig. 3:**
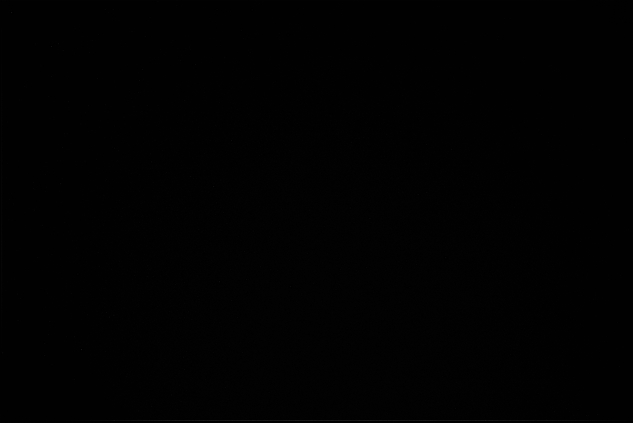
The sample frame for Capture_00088 02_56_08Z.fits after the sharpening process. Please note, the images at this stage of the processing show only the remaining stars and slight haloing against the black background. Although the images in the published dataset are high-resolution, when reduced or compressed it can be hard to make out details at lower zoom levels. The full-size, high-resolution images can be downloaded from the dataset link attached to this article.

Although the background light has been smoothed, and some new information is visible, there are evident problems with the image - primarily, it is overly grey, still has a lot of visible noise, slight vignetting and there is not sufficient contrast. In an attempt to rectify these issues, a further sharpening process was applied to the frames, in which the details and edges of the frame’s information were enhanced as to improve the contrast of visible features. Chao and Tsai^32^ explain that sharpening is a crucial step in astronomical image restoration, as it enhances the fine details that are often important for various forms of inquiry. By augmenting the contrast around the lines and edges of such details, the observable features and structures become more pronounced which improves the ability to accurately interpret the information. For the current data, an unsharp masking technique was applied. As elucidated by Deng^33^, this process is a typical image sharpening tool and involves the application of subsequent processing steps as to enhance the image. Firstly, a Gaussian filter is used to blur the image and therefore create a smoothed version of it in which the high-frequency details such as lines and edges are reduced. Then, the difference between this smoothed image and the original image is computed, with the disparity representing the aforementioned high-frequency components. This essentially creates a mask of the edges and details of the visual information, which is then multiplied by a scale value or sharpening factor - in this case, a relatively standard value of 1.5 - which determines the extent to which the high-frequency information is accentuated in the image. This both improves the contrast of the edges and makes them more prominent. Finally, clipping is once again used to ensure the pixel values are non-negative and fall within the valid range, i.e. 0 to 65535 for 16-bit images such as those in the current dataset, ensuring no pixel underflow or overflow is experienced. This maintains the integrity of the output image and ensures no visual artifacts are introduced by the sharpening process. Following the application of the above steps, output images were generated with the processing code and an example of this is shown in Fig. 3.

Upon reviewing the output images, the processing steps appeared to have fixed a number of the critical issues with the original frames. The background was correctly displaying as solid black, with acceptable contrast against the visible stars and drastically reduced observable vignetting. Furthermore, additional information was now visible in various regions of the image, whilst also retaining the dimmer features that appeared within the span of the original artifact. That being said, as is the case with many astronomical image processing attempts, there are still some notable issues with the images, including slight haloing around more prominent stars and reduced overall image brightness. At this point, the processing steps were applied to the entire dataset, before each frame was converted into a PNG image and stored.

## Data Record

The Frigate dataset^26^ consists of high-resolution astronomical imagery captured by a commercial-grade ground-based optical telescope system operated by ExoAnalytic Solutions Inc. The data include still-frame sequences representing consecutive temporal observations of low Earth orbit (LEO) regions.

The dataset is organised into two primary directories:

- Raw — Contains the original, unprocessed FITS files. These files retain the naming structure and temporal sequence of their source. All files are stored in a single flat directory for ease of access.
- Processed — Contains a subset of the raw images that were suitable for median background subtraction and contrast enhancement. These frames have been converted to PNG format and are named according to their raw counterparts. All files are stored in a single flat directory for ease of access. The first ten and last ten images are absent, as these would not have sufficient adjacent frames for the background subtraction process.

Each FITS file encodes a single still frame and includes a full header containing observational metadata. All metadata are retained in the original FITS headers. The processed directory offers enhanced visual clarity for direct analysis and includes only those frames with sufficient temporal neighbours to enable robust background estimation. This release contains the complete, unabridged raw data archive and is intended to support further research in orbital object detection, astronomical image processing, and machine learning-based surveillance applications.

## Technical Validation

To ensure the usability and technical quality of the Frigate dataset^26^, several key validation steps were performed. The goal was to verify that the raw data files are complete, correctly structured, and scientifically meaningful for downstream analysis in applications such as space domain awareness and orbital object detection.

### FITS File Integrity

All raw FITS files were batch-verified using the astropy.io.fits module to ensure they:

- Opened successfully with no corruption or header parsing errors,
- Contained valid 2D image arrays matching the expected resolution of 9600 × 6422 pixels,
- Included complete and consistent metadata (e.g., DATE-OBS, EXPTIME, BSCALE, BZERO).

Any corrupted or truncated files were omitted from the final release. All retained files conform to standard FITS formatting practices.

### Pixel Intensity Distribution

**Fig. 4:**
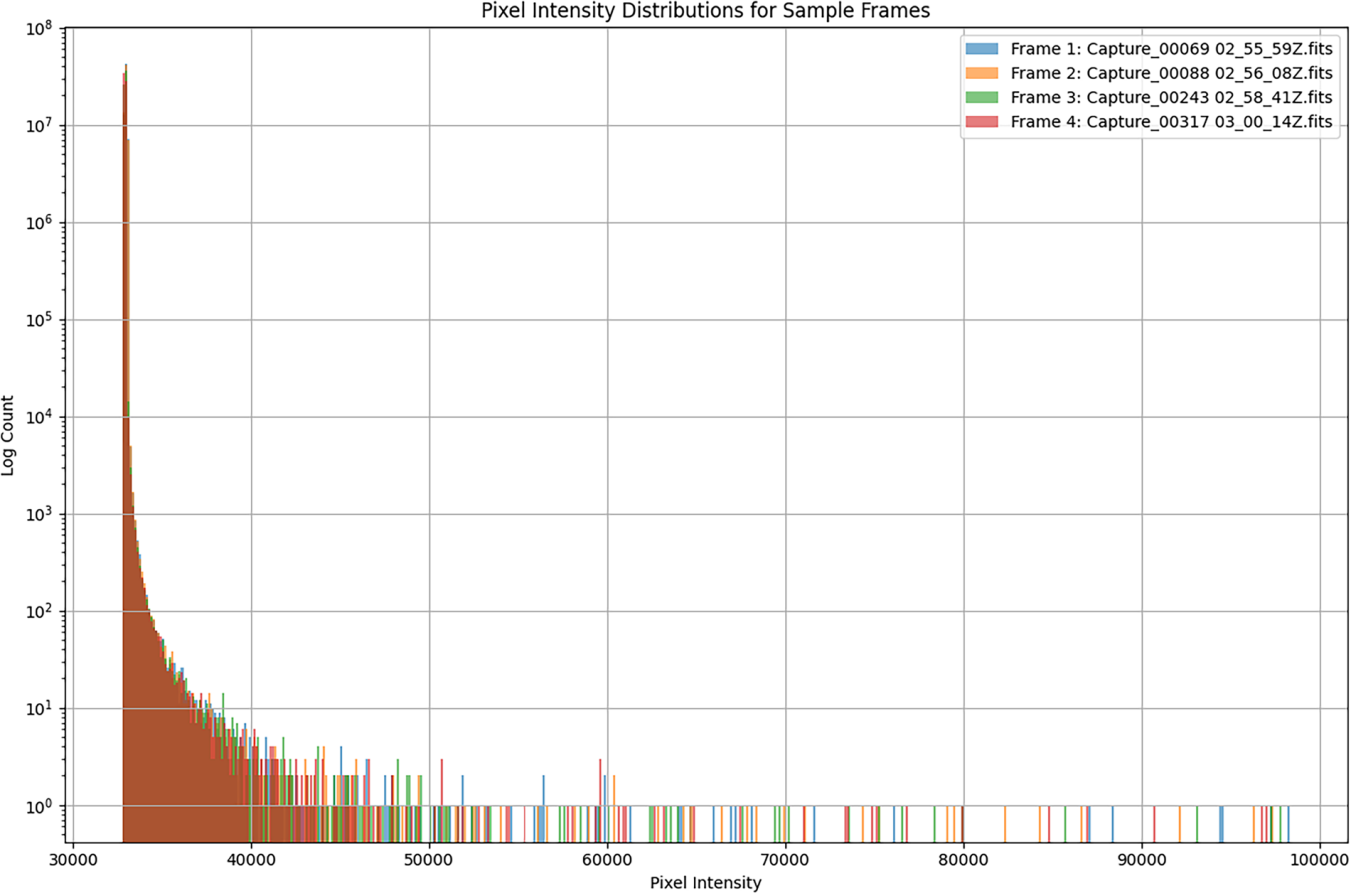
Pixel intensity distributions for sample frames.

To assess image quality and sensor signal fidelity, a pixel intensity histogram was generated for a sample of raw frames. The resulting distributions, shown in Fig. 4, demonstrate consistent and stable structure across different frames in the dataset.

Each histogram exhibits a prominent peak within the lower pixel intensity range (approximately 32,500-33,000), corresponding to the background sky level. The pixel values follow the expected unsigned integer encoding (using BZERO rescaling from FITS format) and are log-distributed, with a long tail representing brighter regions in the frame such as stars or occasional bright artefacts. The high count in the background region confirms that a large proportion of each frame consists of relatively uniform sky background, while the tail reflects the presence of significant image features.

Importantly, the distributions do not appear flat, random or excessively noisy - features that would indicate sensor saturation or corruption. Instead, the structure of the histograms suggests the presence of consistent astronomical signal overlaid with system and atmospheric noise. This aligns with expectations for optical telescope imaging under realistic night-time conditions.

While the known lens artefact is not clearly visible in the histograms, this is due to its broad, diffuse nature. Such artefacts raise pixel values across wide regions of the image in a gradual fashion and are difficult to isolate in 1D histograms. Instead, they manifest as slight skewness or localised intensity increases that become clearer in spatial visualisations or difference maps. Nonetheless, the overall intensity distributions support the technical suitability of the data for downstream analysis tasks such as object detection and background subtraction.

### Temporal Sequence Verification

The dataset is organised chronologically, with each frame representing a consecutive temporal image of the scene. Timestamps in metadata headers were verified to confirm chronological ordering. Some frames are missing from continuous sequences within the dataset. These omissions are believed to result from a combination of technical interruptions (e.g., telescope repositioning or environmental occlusion) and potential redactions by ExoAnalytic Solutions Inc. to protect sensitive observations. No frames were removed during export by the research team, except for a small subset excluded due to insufficient temporal neighbours for reliable background subtraction. This does not materially affect the overall utility or continuity of the dataset.

### Object Detection Confirmation

**Fig. 5:**
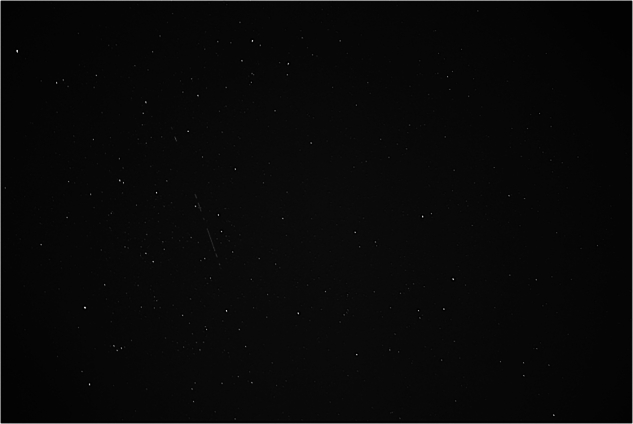
The generated difference image for Capture_01419 03_09_43Z.fits.

In order to evaluate the utility of the dataset with regards to its intended usage for computer vision tasks, an analysis was conducted to attempt to detect objects within the frames. When considering the current dataset, the nature of the images and the scale of the observable region cause some problems. Each image is a single still-frame capture of the LEO region, thus any objects are captured at a specific point in their trajectory. At the scale of the image, an object appears no different to a star in a single frame; it would be impossible to locate an object and draw a bounding box. In order to facilitate detections, by distinguishing any observed objects from the surrounding stars, a decision was made to use difference image analysis. Bramich^34^ explains that this is an astronomical image processing technique that aims to measure the deviations in the objects of frames of the same field at different times. By aggregating the information of a small number of consecutive frames into a single image, the results will barely show the minuscule movement of stars due to the rotation of the earth, yet any transient objects such as satellites that are moving at faster speeds or in varying directions will appear as clearly visible small streaks or lines. For a single frame of the current data^26^ (Capture_01419 03_09_43Z.fits), this was achieved by aggregating the information for the adjacent 25 frames. For each of these frames, the absolute difference between it and the previous frame was calculated and stored, before the overall mean average difference of the sequence was computed by dividing by the number of differences generated. An example of the computed difference image can be seen in Fig. 5.

In the example image, an object can be seen as a visible streak and is clearly discernible from the background. However, this did introduce some minor noticeable visual issues in the form of decreased background contrast and slight vignetting in the corners of the image. Nevertheless, being able to distinguish objects is an important step in confirming the utility of the dataset for use with machine learning algorithms and the generated image offers the starting point for any further processing.

These steps confirm that the dataset is both technically sound and practically usable, even prior to advanced calibration or object annotation. The raw data retain all necessary properties for custom downstream processing depending on the intended research use case.

## Usage Notes

As one of the main potential applications for this dataset is downstream usage within object detection technologies aimed at SSA applications, some further processing work was undertaken and may be prominent for researchers wishing to utilise the data in this domain. In object detection algorithms, bounding boxes and their associated labels play an important role during the training process. A bounding box outlines the region in which an object is wholly located, represented by the coordinates of the box’s *X* and *Y* axis’ centres and its width and height^35^. The algorithm uses the box to identify a focus region in which the features such as edges, textures and pixel intensity can be analysed, confining the learning process to the pixels contained within the boundaries. It learns information by predicting a bounding box on an image, before checking the disparity with the correct ‘ground-truth’ bounding box to measure the accuracy of the prediction. The algorithm then uses this information to minimise a loss function, optimising its predictions and improving its ability to localise on the objects during the training process. In most applications, when training detection algorithms such as YOLO, despite being a lengthy task it can be relatively straightforward to prepare and label the training data once it has been accumulated. Using many commonly available tools, a researcher can enclose the target object in a bounding box and assign a relevant label - it is simple for a human to differentiate a person or an animal from the background, for example.

As the next step for users to complete in preparing the data would be to annotate the images and enclose any visible streaks in bounding boxes, some thought was given to devising methods to optimise this process due to the large number of frames that needed handling. The first idea that formed was to isolate the object streaks by removing the stars from the images, which would allow for the automated annotation of objects. The thought was that if the stars were removed, the background could be again set to solid black and a script could be designed to cycle through each pixel to determine where the intensity changes, thus, where the object begins and ends, and draw the bounding box accordingly. A typical method for removing the stars from astronomical images is the application of a Fourier transform, which is a mathematical tool used to analyse the frequency components of an image^36^. The process generates a magnitude spectrum that reveals the amplitude of these components, indicating how much of each frequency is present, presented on a logarithmic scale that can handle the wide range of possible values. It also generates a phase spectrum, that identifies the position of structures within the image, identifying where the observed frequencies occur. With regards to the current data, the Fourier transform could theoretically be used to separate out the unwanted components, the stars in the image, based on their frequency characteristics. This would be achieved by converting the image from the spatial domain, where the pixels are located, to the frequency domain, where the image is represented by the frequency components. The stars in the images appear as point sources of light, corresponding to high-frequency components in the Fourier domain. Therefore, by filtering out these high-frequency components, it should be possible to apply an inverse Fourier transform and revert to an image with only components that correspond to other information, namely the object streaks. An attempt was made to employ this method to the data. Unfortunately, when reviewing both the generated magnitude and phase spectrums, there was not sufficient disparity between observable signals as to denote those relating to the object or stars exclusively. This is most likely due to both signals being relatively similar and, even when looking at the raw image, the intensity of the object streak is not too dissimilar from many of the stars surrounding it.

**Fig. 6:**
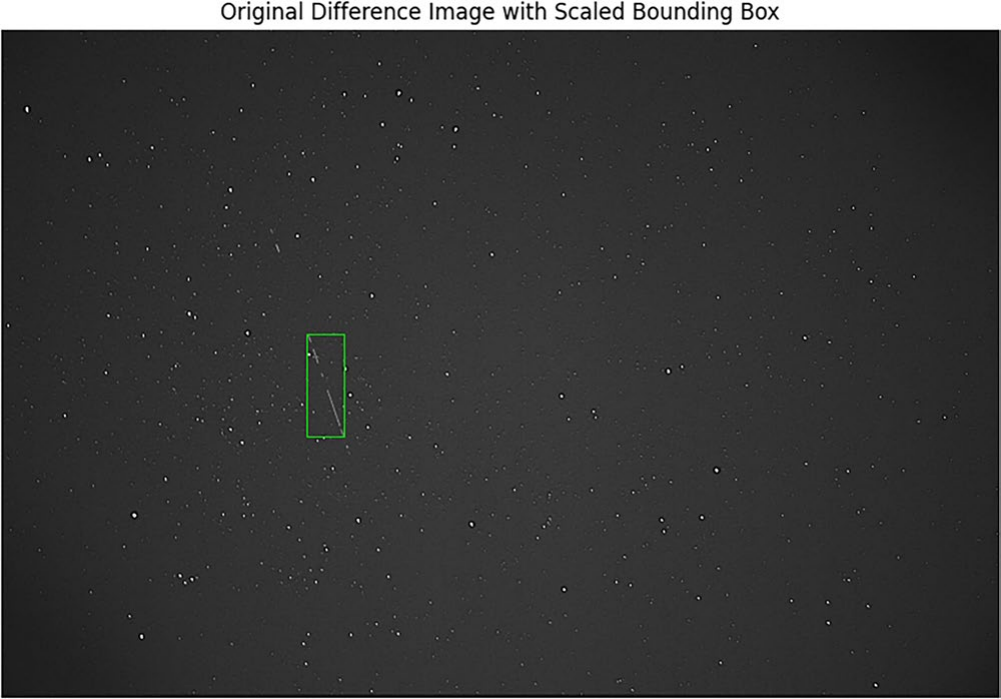
The image displaying the bounded object streak for Capture_01419 03_09_43Z.fits following the automated annotation process.

In order to solve the problem, a new method for automatically annotating the images was considered. This involved using a series of advanced processing techniques to first isolate the object streak as best as possible, then identify its position and use the coordinates to draw a bounding box on the original difference image. The first step in this process was to use the Canny edge detection algorithm^37^, which works by applying a multi-stage process involving Gaussian filtering for noise reduction, gradient calculation to identify edge strength, non-maximum suppression to thin the edges and finally hysteresis thresholding to retain the most valid edges whilst discarding the weak ones. The algorithm’s ability to accurately define edges within noisy images made it a good fit for the current dataset. Following the edge detection, a probabilistic Hough Transform was applied to further detect lines within the detected edges. Kälviäinen *et al*.^38^ explain that this process operates by transforming observed points in the image space into parameter space where the detection of lines is facilitated by the identification of points of intersection. This is beneficial when working with noisy data, as it does not require a perfect or complete line to be present; rather, it probabilistically discriminates segments of lines, which is obviously applicable to the current data as there are missing frames that manifest as visible observation gaps within the difference images. By tuning the parameters of the Hough Transform, the algorithm was able to successfully identify and isolate the object streak in the test image, creating a mask. When applied to the original image, this resulted in all the other information being removed, including the stars, and just leaving the object streak against a black background. The contrast was then adjusted to ensure this was as visible as possible. Next, using the edge detection process, the location of the streak on the masked image was found again, and a padded box was drawn around it. The coordinates of the box were then used to draw a final bounding box on the original difference image, resulting in an automatically annotated object streak. The result of this process on the test image is shown in Fig. 6.

Whilst this process managed to successfully identify the largest portion of the streak, larger gaps in the trajectory were present, indicating the method did struggle to enclose all of the object. Unfortunately this did not seem to be easily solvable, as adjusting the parameters to include the distant parts also resulted in many other star pairings being misidentified as object streaks. Nonetheless, this process offers a starting point for users of the dataset to begin processing the data for object detection pipelines and all code for this will be supplied alongside the data.

## Data Availability

The Frigate dataset is openly available via Figshare at 10.6084/m9.figshare.29545667.

## Code availability

Custom Python code was developed to preprocess and visualise the raw FITS files within the Frigate dataset. All scripts used for dataset preparation, including those used to generate the processed PNG versions of the raw files and the figures presented in this manuscript, are available via a public GitHub repository (https://github.com/DanSRoll/frigate). The software environment was managed using Anaconda, with Python 3.10. The code is provided under an open-source license to ensure reproducibility and transparency in data preparation workflows.
